# Effect of enzyme therapy and prognostic factors in 69 adults with Pompe disease: an open-label single-center study

**DOI:** 10.1186/1750-1172-7-73

**Published:** 2012-09-26

**Authors:** Juna M de Vries, Nadine AME van der Beek, Wim CJ Hop, Francois PJ Karstens, John H Wokke, Marianne de Visser, Baziel GM van Engelen, Jan BM Kuks, Anneke J van der Kooi, Nicolette C Notermans, Catharina G Faber, Jan JGM Verschuuren, Michelle E Kruijshaar, Arnold JJ Reuser, Pieter A van Doorn, Ans T van der Ploeg

**Affiliations:** 1 Department of Neurology & Center for Lysosomal and Metabolic Diseases, Erasmus MC University Medical Center, Rotterdam, the Netherlands; 2 Department of Pediatrics & Center for Lysosomal and Metabolic Diseases, Erasmus MC University Medical Center, Rotterdam, the Netherlands; 3 Department of Epidemiology and Biostatistics, Erasmus MC University Medical Center, Rotterdam, the Netherlands; 4 Department of Internal Medicine & Center for Lysosomal and Metabolic Diseases, Erasmus MC University Medical Center, Rotterdam, the Netherlands; 5 Department of Neurology, Rudolf Magnus Institute of Neurosciences, University Medical Center Utrecht, Utrecht, the Netherlands; 6 Department of Neurology, Academic Medical Center, Amsterdam, the Netherlands; 7 Department of Neurology, Radboud University Nijmegen Medical Center, Nijmegen, the Netherlands; 8 Department of Neurology, University Medical Center Groningen, Groningen, the Netherlands; 9 Department of Neurology, Maastricht University Medical Center, Maastricht, the Netherlands; 10 Department of Neurology, Leiden University Medical Center, Leiden, the Netherlands; 11 Department of Clinical Genetics & Center for Lysosomal and Metabolic Diseases, Erasmus MC University Medical Center, Rotterdam, the Netherlands

**Keywords:** Pompe disease, Glycogen storage disease type II, OMIM number 232300, Acid α-glucosidase, Alglucosidase alfa, Enzyme replacement therapy, Lysosomal storage disorder, Muscle strength, Lung function

## Abstract

**Background:**

Enzyme replacement therapy (ERT) in adults with Pompe disease, a progressive neuromuscular disorder, is of promising but variable efficacy. We investigated whether it alters the course of disease, and also identified potential prognostic factors.

**Methods:**

Patients in this open-label single-center study were treated biweekly with 20 mg/kg alglucosidase alfa. Muscle strength, muscle function, and pulmonary function were assessed every 3–6 months and analyzed using repeated-measures ANOVA.

**Results:**

Sixty-nine patients (median age 52.1 years) were followed for a median of 23 months. Muscle strength increased after start of ERT (manual muscle testing 1.4 percentage points per year (pp/y); hand-held dynamometry 4.0 pp/y; both p < 0.001). Forced vital capacity (FVC) remained stable when measured in upright, but declined in supine position (−1.1 pp/y; p = 0.03). Muscle function did not improve in all patients (quick motor function test 0.7 pp/y; p = 0.14), but increased significantly in wheelchair-independent patients and those with mild and moderate muscle weakness.

Relative to the pre-treatment period (49 patients with 14 months pre-ERT and 22 months ERT median follow-up), ERT affected muscle strength positively (manual muscle testing +3.3 pp/y, p < 0.001 and hand-held dynamometry +7.9 pp/y, p < 0.001). Its effect on upright FVC was +1.8 pp/y (p = 0.08) and on supine FVC +0.8 (p = 0.38). Favorable prognostic factors were female gender for muscle strength, and younger age and better clinical status for supine FVC.

**Conclusions:**

We conclude that ERT positively alters the natural course of Pompe disease in adult patients; muscle strength increased and upright FVC stabilized. Functional outcome is probably best when ERT intervention is timely.

## Background

Pompe disease (OMIM number 232300) is an autosomal recessive metabolic myopathy caused by deficiency of the lysosomal enzyme acid α-glucosidase. This deficiency impairs lysosomal glycogen breakdown, leading to glycogen accumulation in several tissues [1-4]. The disease covers a broad clinical spectrum, ranging from a rapidly progressive infantile phenotype that results in death within the first year of life, to more slowly progressive forms in children and adults [5-11]. In adults, the disease generally presents as a limb-girdle myopathy. As well as the skeletal muscles, respiratory muscles – including the diaphragm – are affected [1,12,13]. As the disease progresses, most patients lose ambulation and require ventilatory support [5,10,11,14,15].

Although Pompe disease used to be untreatable, patients’ prospects changed in 2006 upon the introduction of enzyme replacement therapy (ERT) with recombinant human acid α-glucosidase. Initial studies in infants showed that ERT improved survival and motor outcome [16-23]. Several studies focusing on adult patients have been published since registration, but most report data in relatively small numbers of patients, or have a short follow-up [24-29]. Proof of efficacy was provided by the 18-month randomized-placebo controlled trial in 90 patients, 60 of whom received alglucosidase alfa. This showed that walking distance improved and pulmonary function in upright position stabilized [29]. As mild and severely affected patients had been excluded, the trial involved a selected group of patients.

The aims of the current study were therefore 1) to determine whether ERT alters the progressive course of Pompe disease in a broader adult patient population ranging from very severely affected to mildly affected; 2) to determine how much ERT alters the course of the disease relative to that reflected in pre-treatment data; and 3) to identify prognostic factors for response to treatment.

## Methods

### Patients and study design

This single-center, prospective, open-label cohort study on the use of ERT was conducted from January 2005 to August 2009 at the Center for Lysosomal and Metabolic Diseases, Erasmus MC University Medical Center, Rotterdam, which is the Dutch national referral center for Pompe patients.

Patients were eligible for inclusion if 1) they were over 18 years of age; 2) their diagnosis had been confirmed by enzyme analysis in leukocytes or fibroblasts [30-32] and by mutation analysis; [33] 3) they had not previously been treated with recombinant human acid α-glucosidase; 4) they had been treated for a minimum of 5 months; and 5) they were symptomatic, i.e. had measurable muscle weakness and/or diminished pulmonary function.

Patients received intravenous infusions with 20 mg/kg alglucosidase alfa every two weeks. Clinical assessments were performed every three to six months before the start of ERT and every three months thereafter.

The study protocol was approved by the Medical Ethical Committee at Erasmus MC University Medical Center. All patients provided written informed consent.

Twenty of the 69 patients participated in the randomized-placebo controlled trial on the safety and efficacy of alglucosidase alfa in late-onset Pompe disease; 13 in the treatment arm and seven in the placebo arm [29]. Data on FVC in upright position of these patients collected during the 18 months study period were included in the current analyses.

### Skeletal muscle strength and function

Skeletal muscle strength was measured by manual muscle testing using the Medical Research Council (MRC) grading scale (range 0–5) and hand-held dynamometry (HHD) (Cytec dynamometer, Groningen, the Netherlands) [34,35]. The following muscle groups were tested: neck extensors, neck flexors, shoulder abductors, elbow flexors, elbow extensors, hip flexors, hip abductors, knee flexors, and knee extensors. The MRC grade was also assessed for shoulder adductors, exorotators and endorotators, hip extensors, and hip adductors. An MRC sumscore was derived by adding the grades for all 26 muscles and expressing the sum as a percentage of the maximum possible score. HHD values (Newton) of each muscle group were first expressed as a percentage of the median strength of healthy males or females, [34] and then combined into a sumscore by averaging these for all 16 muscle groups. If values for three or more muscle groups were missing, no sumscores were calculated for either method.

Muscle function was assessed using the Quick Motor Function Test (QMFT); [36] this consists of 16 motor skills related to daily activities that require the use of muscles of the shoulder girdle, trunk, pelvic girdle and/or proximal lower limbs. Each item was scored on a 5-point ordinal scale, with 0 representing ‘cannot perform task’ and 4 ‘can perform task with no effort’. Adding all items gives a total score between 0 and 64. The actual sumscore was expressed as a percentage of the maximum score.

The use of wheelchair and walking aids was registered at each visit.

### Pulmonary function

Forced vital capacity (FVC) in upright and supine positions was measured using spirometry as described earlier [13,37]. Results were expressed as a percentage of the predicted normal value [38,39]. Values lower than 80% of predicted normal values were considered to be abnormal. The use of ventilatory support and hours of use per day was registered at each visit.

### Safety assessments and laboratory investigations

Vital signs and adverse events were recorded at each visit. Electrocardiograms, a physical examination, and hematological, biochemical and urine analyses were made at regular intervals.

### Statistical analysis

Longitudinal analysis of the outcome parameters (MRC, HHD and QMFT sumscores and FVC in upright and supine positions) was performed using repeated-measures ANOVA (random coefficient models). This method allows for irregular measurement times and different treatment durations. First, we assessed the mean annual change in the outcome parameters during the treatment period. Mean annual changes were expressed in absolute percentage points (pp/y). Analyses were also stratified by subgroups described in previous studies: gender, age (<45 years and ≥45 years old), disease duration (<15 years and ≥15 years), wheelchair use, ventilation use, FVC in upright position (≥80% and <80%) and MRC sumscore (in tertiles) [13,29].

Second, for the patients with pre-treatment and treatment data we established the extent to which the course of disease altered after starting ERT. To be included in this analysis, patients should have had at least one measurement done a minimum of 4 months before ERT. We included a linear effect of time before and during ERT in the repeated-measures ANOVA. Per individual, the two linear segments connect with each other at the time of start of ERT. This method is also known as the “broken stick method” or as “piece-wise linear regression”.

Third, we investigated prognostic factors for treatment response. As well as performing the subgroup analysis, we assessed the association between patients’ characteristics and their individual response to ERT. Patients were classified into three groups: 1) non-responders, i.e. people in whom the course of disease (measured by one of the outcome parameters) was the same or worse during ERT than before; 2) good responders, whose disease course improved more than the median improvement of responders; and 3) moderate responders. Each patient’s response was represented by the individual change in regression lines calculated in the “broken stick” analysis. In patients without pre-treatment data, the natural course slope was imputed. Associations were tested using the Spearman test for continuous variables and the chi-square trend test for categorical variables.

Analyses were performed with SPSS for Windows (version 17, SPSS Inc., Chicago, IL) or SAS (version 9.2, SAS Institute Inc., Cary, NC). A p-value lower than or equal to 0.05 (two-sided) was considered significant.

## Results

### Patients

In total, 71 adult Pompe patients started ERT within the study period. Two were excluded because they had received ERT for less than five months: one died two months after starting ERT due to a dissection of the aortic arch; the second withdrew from the study after four months and died six months later due to respiratory insufficiency.

Table 1 shows the patients’ characteristics at start of ERT. The median age at the start of ERT was 52.1 years, 52% were male, 40% used a wheelchair, and 37% used mechanical ventilation. The median treatment duration was 23 months (range 5 – 47 months). Of the 69 patients, 13 were treated for more than 3 years and six patients for less than 1 year, respectively for 5, 6, 7, 10, and two for 11 months.

**Table 1:** Patients’ characteristics and baseline values of clinical outcome measures at start of ERT

**Characteristic**	**Total study population (N = 69)**	**Patients with pre-ERT and ERT follow-up (N = 49)**	P-value*
Age at start of ERT in years – median (range)	52.1 (26.2 – 76.3)	50.1 (26.2 – 74.0)	0.83
Age at onset of symptoms in years – median (range)	30.8 (1.4 – 62.0)	32.0 (1.4 – 62.0)	0.63
Age at diagnosis in years – median (range)	39.6 (1.4 – 63.8)	40.9 (1.4 – 63.0)	0.97
Duration of disease at start of ERT in years – median (range)	9.3 (0.2 – 31.2)	8.3 (0.5 – 31.2)	0.74
MRC sumscore at start of ERT in percentage – median (range)	77.7 (48.3 – 92.3)	79.2 (60.8 – 92.3)	0.52
HHD sumscore at start of ERT in percentage – median (range)	69.3 (25.9 – 94.1)	69.3 (25.9 – 94.1)	0.97
QMFT score at start of ERT in percentage – median (range)	55.6 (10.0 – 89.1)	59.5 (21.7 – 89.1)	0.42
Time of mechanical ventilation at start of ERT per day in hours – median (range)	11.5 (0.0 –24.0)	10.5 (0.0 –24.0)	0.81
FVC in upright position at start of ERT in percentage – median (range)	68.3 (11.3 – 106.9)	71.9 (26.5 – 106.9)	0.43
FVC in supine position at start of ERT in percentage – median (range)	46.8 (23.0 – 99.0)	52.4 (23.0 – 99.0)	0.71
Gender – No. of patients (%)			0.43
- Male	36 (52)	21 (43)	
- Female	33 (48)	28 (57)	
Wheelchair at start of ERT – No. of patients (%)			
- No wheelchair use	42 (61)	33 (67)	0.22
- Partial wheelchair use	8 (12)	8 (16)	
- Permanent wheelchair use	19 (28)	8 (16)	
Mechanical ventilation at start of ERT – No. of patients (%)			
- No mechanical ventilation	44 (64)	36 (74)	0.72
- Non-invasive mechanical ventilation	19 (28)	10 (20)	
- Invasive mechanical ventilation	6 (9)	3 (6)	

*ERT* = Enzyme Replacement Therapy; *MRC* sumscore = Medical Research Council sumscore; *HHD* sumscore = Hand-Held Dynamometry sumscore; *QMFT* score = Quick Motor Function Test score; *FVC* = Forced Vital Capacity; *N/No*. = Number of patients.

* Differences between the total group (N = 69) and the group with pre-ERT and ERT follow-up (N = 49) were calculated using chi-square tests for the categorical variables, and using Mann–Whitney tests for continuous variables.

All but one patient carried the common c.-32-13 T > G (IVS1-13 T > G) mutation on one allele in combination with another pathogenic mutation on the second allele. Of the patients carrying the c.-32-13 T > G (IVS1-13 T > G) mutation, 14 different mutations were found on the second allele. Forty-eight percent carried the c.525 delT and 16% the c.2481 + 102-2646 + 31del mutation. The other mutations found were the c.-32-13 T > G + c.1076-22 T > G, c.1115A > T, c.1396 G > T, c.1548 G > A, c.172C > T, c.1799 G > A, c.2314 T > C, c.378_379del, c.461_469del, c.701C > A, c.896 T > C and c.925 G > A mutation. Of those mutations, 74% was indicated as very severe and 26% as potentially less severe (http://www.pompecenter.nl). For three patients the result of the mutation analysis could not be tracked, because these were performed by another center.

For 49 patients, pre-treatment data were available in addition to the treatment follow-up. These were slightly younger and less severely affected. Their median follow-up was 14 months before (range 4–33), and 22 months during ERT (range 6–41).

### Skeletal muscle strength

During ERT, the MRC sumscore among all 69 patients rose by an average of 1.4 pp/y, and the HHD sumscore by 4.0 pp/y (both p < 0.001; Table 2). All individual muscle groups contributed to the effect (p *≤* 0.02 for all muscle groups). Subgroup analyses showed that the mean annual increase in muscle strength during ERT was greater for women than for men (2.6 pp/y vs. 0.4 pp/y; difference between groups p < 0.001 for MRC; and 6.3 pp/y vs. 2.0 pp/y, difference between groups p = 0.05 for HHD). For the other subgroups investigated, there were no significant differences in the increase in muscle strength during ERT.

**Table 2:** Clinical outcome measures during enzyme replacement therapy and relative to the natural course of disease

**Clinical Outcome Measure**	**Natural course mean pp/y (95% CI) P-value**		**Treatment course mean pp/y (95% CI) P-value**		Difference* mean pp/y (95% CI) P- value	
MRC sumscore *Total study population (N = 69, M = 558)*			1.4 (0.8 to 2.1)	<0.001		
MRC sumscore*Patients with pre-ERT + ERT follow-up (N = 49, M = 523)*	−1.2 (−2.1 to −0.4)	0.006	2.1 (1.2 to 3.0)	<0.001	3.3 (1.9 to 4.7)	<0.001
HHD sumscore *Total study population (N = 64, M = 503)*			4.0 (2.5 to 5.6)	<0.001		
HHD sumscore *Patients with pre-ERT + ERT follow-up (N = 42, M = 435)*	−2.8 (−4.2 to −1.3)	<0.001	5.1 (3.0 to 7.3)	<0.001	7.9 (5.0 to 10.7)	<0.001
QMFT score *Total study population (N = 69, M = 553)*			0.7 (−0.2 to 1.7)	0.14		
FVC in upright position *Total study population (N = 62, M = 475)*			0.1 (−1.0 to 1.1)	0.92		
FVC in upright position *Patients with pre-ERT + ERT follow-up (N = 46, M = 480)*	−2.0 (−3.1 to −0.8)	0.001	−0.2 (−1.6 to 1.2)	0.76	1.8 (−0.2 to 3.7)	0.08
FVC in supine position *Total study population (N = 54, M = 411)*			−1.1 (−2.1 to −0.1)	0.03		
FVC in supine position *Patients with pre-ERT + ERT follow-up (N = 42, M = 436)*	−1.8 (−2.9 to −0.7)	0.002	−1.0 (−2.3 to 0.3)	0.12	0.8 (−0.9 to 2.4)	0.38

*ERT* = Enzyme Replacement Therapy; pp/y = percentage points per year; 95% CI = 95% Confidence Interval; *MRC sumscore* = Medical Research Council sumscore; *QMFT score* = Quick Motor Function Test score; *HHD sumscore* = Hand-Held Dynamometry sumscore; *FVC* = Forced Vital Capacity; *N* = Number of patients; *M* = Number of Measurements. Data shown are mean changes in percentage points per year as calculated by repeated-measures ANOVA.

* Difference between the course of disease during treatment and the natural course.

**Figure 1:**
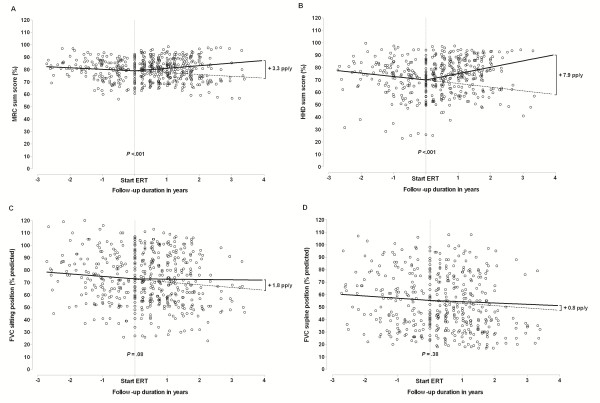
**The clinical course of disease before and after the start of enzyme replacement therapy (ERT) in the 49 patients with ERT and pre-ERT follow-up data for A) MRC sumscore; B) hand-held dynamometry (HHD) sumscore; C) forced vital capacity (FVC) in upright position; and D) FVC in supine position.** The figure shows all the individual observations for the different outcome measures (shown as dots) and the solid lines represent the estimated mean trend in these observations as calculated by the ‘broken stick’ repeated-measures ANOVA. The dotted lines represent the extrapolated natural course slopes.

Before the 49 patients with pre-treatment and treatment data started ERT, their muscle-strength sumscores declined significantly (−1.2 pp/y for MRC, p = 0.006; –2.8 pp/y for HHD, p < 0.001). Treatment data produced a significant improvement, the differences being +3.3 pp/y for the MRC sumscore and +7.9 pp/y for the HHD sumscore (both p *<* 0.001; Table 2, Figure 1A and B).

### Skeletal muscle function

Although QMFT scores for all 69 patients increased by an average of 0.7 pp/y during ERT, this was not significant (p *=* 0.14). Subgroup analyses showed that while muscle function improved in patients with mild muscle weakness (2.1 pp/y, p = 0.01) and moderate muscle weakness (1.6 pp/y, p = 0.05), it fell by 1.4 pp/y (p = 0.08) in patients with severe muscle weakness (difference between groups p = 0.004). In line with this, QMFT scores rose in wheelchair-independent patients (1.7 pp/y, p = 0.008), but did not improve in those who were wheelchair-dependent (−0.6 pp/y, p = 0.39; difference between groups p = 0.02).

The number of patients who were partially or fully wheelchair dependent at the last follow-up visit was the same as at the start of ERT. Nevertheless, two of the 27 patients who used walking aids at the start of ERT regained the ability to walk independently during ERT. Two other patients became dependent on walking aids.

### Pulmonary function and use of mechanical ventilation

During treatment with ERT, FVC in upright position remained stable (0.1 pp/y, p = 0.92), while FVC in supine position declined (−1.1, p = 0.03) (Table 2). Subgroup analyses showed that FVC in supine position did remain stable in patients under 45 years old (0.0 pp/y, p = 1.0), but declined in those 45 years and over (−2.1 pp/y, p = 0.002) (difference between groups p = 0.03). There were no differences between subgroups for FVC in upright position.

Before start of ERT, FVC in upright and supine positions both declined significantly (−2.0 pp/y, p = 0.001 and −1.8 pp/y, p = 0.002, respectively). Compared to this, FVC in upright position improved during ERT, albeit at borderline statistical significance (+1.8 pp/y, p *=* 0.08), while FVC in supine position did not (+0.8, p = 0.38) (Table 2 and Figure 1C and D).

For the whole group there was no change in the median number of hours of ventilation per day between the start of ERT and the last treatment visit (Wilcoxon signed-rank test p = 0.88). Mechanical ventilation time could be reduced by one or more hours per day in nine of the 25 patients who had been using mechanical ventilation at start of ERT. One of these was able to discontinue the use of his ventilator. In 14 patients, ventilation times remained the same, while ventilation was intensified in two. Nocturnal ventilation was initiated in two others.

### Individual response with regard to skeletal muscle strength and pulmonary function

**Figure 2:**
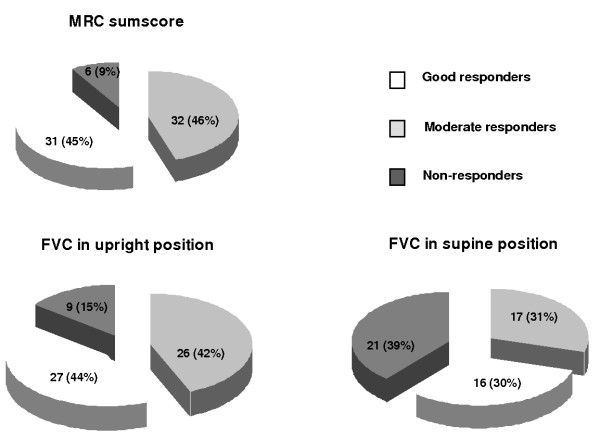
**Individual response groups for skeletal muscle strength and pulmonary function.** The number and percentage of good responders, moderate responders and non-responders are shown for MRC sumscore, and forced vital capacity (FVC) in upright and supine positions.

With regard to MRC sumscore, 31 patients (45%) were good responders, 32 (46%) moderate, and six (9%) non-responders (Figure 2). More women than men were good responders (p = 0.002, Table 3). Twenty-seven of the 62 patients with FVC data in upright position (44%) responded well, 26 (42%) moderately, and nine (15%) poorly. The characteristics between these FVC responder categories did not differ significantly.

**Table 3:** Characteristics of individual response groups with regard to skeletal muscle strength and pulmonary function

	**Non-responders**	**Moderate Responders**	**Good responders**	**P-value**
**Response groups for MRC sumscore – No. of patients (%)**	**6 (9)**	**32 (46)**	**31 (45)**	
Female – No. of patients (%)	*1 (17)*	*11 (34)*	*21 (68)*	*0.002*
Age at start of ERT in years – median (range)	51.6 (38.0 – 66.2)	50.0 (26.2 – 67.0)	52.7 (29.2 – 76.3)	0.47
Disease duration at start of ERT in years – median (range)	12.1 (3.6 – 22.7)	11.1 (0.9 – 31.2)	9.0 (0.2 – 29.1)	0.55
MRC sumscore at start of ERT in percentage – median (range)	80.8 (60.8 – 86.9)	80.0 (48.3 – 91.5)	74.6 (53.3 – 92.3)	0.12
Wheelchair use at start of ERT – No. of patients (%)	2 (33)	9 (28)	16 (52)	0.11
FVC in upright position at start of ERT in percentage – median (range)	61.5 (34.4 – 79.3)	69.9 (11.3 – 105.0)	69.3 (16.4 – 106.9)	0.48
Ventilation use at start of ERT – No. of patients (%)	1 (17)	12 (38)	12 (39)	0.49
**Response groups for FVC in upright position – No. of patients (%)**	**9 (15)**	**26 (42)**	**27 (44)**	
Female – No. of patients (%)	5 (56%)	14 (54%)	13 (48%)	0.64
Age at start of ERT in years – median (range)	52.6 (31.9 – 69.2)	51.2 (35.7 – 71.7)	43.3 (26.2 – 76.3)	0.16
Disease duration at start of ERT in years – median (range)	8.3 (0.6 – 31.2)	14.1 (0.9 – 27.0)	4.7 (0.2 – 29.1)	0.06
MRC sumscore at start of ERT in percentage – median (range)	74.6 (63.1 – 91.5)	77.7 (48.3 – 90.0)	80.8 (67.5 – 92.3)	0.26
Wheelchair use at start of ERT – No. of patients (%)	4 (44%)	10 (38%)	6 (22%)	0.15
FVC in upright position at start of ERT in percentage – median (range)	66.6 (51.4 – 100.5)	68.3 (11.3 – 106.9)	69.9 (16.4 – 105.0)	0.95
Ventilation use at start of ERT – No. of patients (%)	2 (22)	8 (31)	8 (30)	0.76
**Response groups for FVC in supine position – No. of patients (%)**	**21 (39)**	**17 (31)**	**16 (30)**	
Female – No. of patients (%)	10 (48)	10 (59)	10 (63)	0.36
Age at start of ERT in years – median (range)	*51.7 (31.9 – 67.0)*	*47.9 (26.2 – 62.9)*	*40.4 (29.2 – 74.0)*	*0.007*
Disease duration at start of ERT in years – median (range)	10.7 (0.6 – 31.2)	4.7 (0.5-21.0)	5.0 (0.9 – 29.1)	0.12
MRC sumscore at start of ERT in percentage – median (range)	*76.9 (60.8 – 91.5)*	*80.8 (69.2 – 90.8)*	*81.9 (69.2 – 92.3)*	*0.02*
Wheelchair use at start of ERT – No. of patients (%)	*8 (38)*	*2 (12)*	*2 (13)*	*0.05*
FVC in upright position at start of ERT in percentage – median (range)	*66.6 (45.4 – 106.9)*	*65.7 (41.5 – 105.8)*	*81.0 (62.6 – 105.0)*	*0.02*
Ventilation use at start of ERT – No. of patients (%)	*4 (19)*	*6 (35)*	*0 (0)*	*0.01*

*ERT* = Enzyme Replacement Therapy; *MRC* sumscore = Medical Research Council sumscore; *FVC* = Forced Vital Capacity; *No.* = Number of patients.

Although FVC in supine position declined in the whole group during ERT, almost two-thirds of patients improved after starting ERT: 16 of 54 patients (30%) responded well, 17 (31%) moderately and 21 (39%) did not respond (Figure 2). Better responses were associated with younger age (p = 0.007), less severe muscle weakness (p = 0.02), wheelchair independence (p = 0.05) and better pulmonary function in upright position (p = 0.02) (Table 3). None of the good responders were dependent on artificial ventilation.

Responses for muscle strength correlated poorly with responses for FVC. The correlation between response categories for FVC in upright and supine was moderate (Spearman’s ρ = 0.56, p <0.001).

### Safety assessments and laboratory investigations

Laboratory safety parameters and ECGs remained unchanged during ERT. In total, 12 patients (17%) developed one or more infusion-associated reaction (IARs). These were similar to the IARs described in the randomized-placebo controlled study; most could be controlled by slowing infusion rates and/or giving premedication [29]. To prevent further IARs, seven patients received antihistamines as pre-medication, and five a combination of antihistamines and corticosteroids. At the end of the study three patients still had IARs, three patients were using antihistamines as premedication and two a combination of corticosteroids and antihistamines. Enzyme replacement therapy was discontinued in three patients who experienced IARs. In only one patient this was for safety reasons: this patient had a medical history of multiple auto-immune diseases and drug-induced allergies. In one of the other two patients IARs co-occurred with a very high antibody titer and a poor response to ERT, as described previously [40].

Two severely affected patients died. Their causes of death (sepsis after severe decubitus and chronic respiratory insufficiency) were considered to be unrelated to ERT.

## Discussion

This study describes a large cohort of adult Pompe patients receiving treatment with alglucosidase alfa. It reflects a unique situation in which most patients were also prospectively followed before starting therapy, thereby extending median follow-up to 3 years (14 months before starting ERT and 23 months afterwards). We found that ERT significantly altered the natural course of disease in adult Pompe patients. Muscle strength increased significantly after they started ERT, and FVC in upright position stabilized. Even though, at group level, FVC in supine position and muscle function did not improve during ERT, there were improvements in certain subgroups of patients.

Like previous studies, we found that muscle strength deteriorated significantly before the start of therapy [10,11] However, the improvement in muscle strength after the start of ERT was greater than that reported in other studies [25-27,29,41]. There may be various reasons for this. Our study was restricted to adult patients, but included patients across the entire disease spectrum. We tested more muscle groups, included more patients, and followed a longer treatment period than other studies, thereby producing over 500 measurements in total.

One new finding of this study is that women benefit more from ERT with respect to muscle strength than males. At the same dosage of 20 mg/kg bodyweight, it is possible that the relative dose of alglucosidase alfa received per gram of muscle-fiber tissue is higher in women than in men. Men generally have a higher lean body mass than women, and thus a somewhat higher muscle mass per kg [42]. As women also have smaller muscle fibers than men, they have a higher ratio of muscle-fiber surface to muscle-fiber volume. Consequently, they may have relatively more mannose 6-phosphate surface receptors, which mediate the uptake of alglucosidase alpha [42,43]. Other factors that may underlie the greater benefit women derive from ERT include muscle-fiber types, activity patterns, and hormonal influences.

Our study did not incorporate the six-minute walk test, a measure of functional endurance used in other studies [24,27,29]. Instead, we assessed motor skills related to daily activities, using the QMFT, which was recently validated for use in patients with Pompe disease [36]. Although there was no change in muscle function across the entire group, there were significant improvements in wheelchair-independent patients and those with less pronounced muscle weakness. This finding indicates that timely intervention with ERT may be crucial to improving muscle function. The same is suggested by the results of the subgroup analysis in the trial of late-onset patients [29].

The stabilization of FVC in upright position in our patients was similar to that recorded in the trial and in other studies; [24-27,29] the decline in FVC before ERT was similar to that observed in the placebo arm of the trial and natural course studies [10,11,13,29].

This is the first study to report on the effect of ERT on the FVC in supine position. In the whole study population, this measure continued to deteriorate despite ERT, but individual results showed an improvement in almost two-thirds of patients. Patients were more likely to improve if they were younger, were independent of artificial ventilation, had a better FVC in upright position, and had less severe muscle weakness at the start of treatment. Again, this suggests that starting ERT early in the disease course may be beneficial.

Because ERT has been available since 2006, we performed an open-label study rather than a clinical trial, assessing the effect of ERT in all adult patients – from mild to severely affected – for many of whom we had also collected pre-treatment data prospectively. As this is an observational study, we could not correct for residual confounding. The small sample size inherent to rare disorders meant that we could not apply a full multivariate model to identify prognostic factors.

## Conclusions

In summary, by improving muscle strength and stabilizing pulmonary function in upright position, treatment with alglucosidase alfa positively altered the natural course of Pompe disease in adults. As well as finding that female gender is potentially a favorable prognostic factor for the effect of ERT on muscle strength, we found that younger age and better clinical status are favorable prognostic factors for pulmonary function. This suggests that it is important to start treatment early in the course of disease. Prognostic variables may help to identify patients with the best chances of benefiting from treatment.

## Competing interests

Research on Pompe disease at Erasmus MC is financially supported by the Erasmus MC Revolving Fund [project number 1054, NAMEvdB]; European Union, 7^th^ Framework Programme “Euclyd – a European Consortium for Lysosomal Storage Diseases” [health F2/2008 grant number 201678]; ZonMw – Netherlands organization for health research and development [grant number 152001005]; and the Princess Beatrix Fund [project number OP07-08]. Since August 2004, ATvdP and AJJR have provided consulting services for Genzyme Corp, Cambridge, MA, USA, under an agreement between Genzyme Corp and Erasmus MC University Medical Center, Rotterdam, the Netherlands.

## Authors’ contributions

JMV and NAMEB participated in the design of the study, carried out the assessments, performed the statistical analyses and drafted the manuscript. WCJH participated in the design of the study and supervised the statistical analysis. FPJK, JHW, MV, BGME, JBMK, AJK, NCN, CGF, JJGMV, were involved in the setup of the study, data interpretation, and critically reviewed the paper, MEK contributed to the analyses and interpretation of the data and revised the manuscript, AJJR, PAD and ATP conceived of the study, participated in its design and interpretation and coordinated the drafting of the manuscript. All authors read and approved the final manuscript.
